# Does Exposure of Broodstock to Dietary Soybean Meal Affect Its Utilization in the Offspring of Zebrafish (*Danio rerio*)?

**DOI:** 10.3390/ani12121475

**Published:** 2022-06-07

**Authors:** Karolina Kwasek, Samuel Patula, Michal Wojno, Frank Oliaro, Chrissy Cabay, Lee J. Pinnell

**Affiliations:** 1 Center for Fisheries, Aquaculture, and Aquatic Sciences, Southern Illinois University, 1125 Lincoln Dr. Life Science II, Room 251, Carbondale, IL 62901, USA; patulasam@gmail.com (S.P.); michal.wojno@siu.edu (M.W.); 2 A. Watson Armour III Center for Animal Health and Welfare, John G. Shedd Aquarium, Chicago, IL 60605, USA; foliaro@sheddaquarium.org (F.O.); ccabay@sheddaquarium.org (C.C.); 3 Veterinary Education, Research, and Outreach Program, Texas A&M University, Canyon, TX 79015, USA; ljpinnell@gmail.com

**Keywords:** gut microbiome, nutritional programming, soybean meal, zebrafish

## Abstract

**Simple Summary:**

Replacement of fishmeal in fish diets with plant protein has been an ongoing challenge. High-quality plant protein concentrates are widely used since their digestibility can be comparable to fishmeal. However, their price can exceed the cost of marine raw materials. Progress with utilization of lower-quality plant protein sources has been made but a number of concerns must be overcome to maintain acceptable growth rates at high fishmeal substitution levels. Nutritional programming represents a promising approach to offset the negative effects of dietary plant protein through its exposure in early life. We tested an unconventional programming strategy by exposing parental zebrafish to soybean meal diet to improve dietary soybean meal utilization in progeny fish. The study observed a strong trend showing better growth performance between progeny zebrafish fed soybean meal diet that originated from broodstock exposed to soybean meal as opposed to progeny fish fed soybean meal diet that originated from fishmeal diet fed broodstock. However, the study found no changes in the richness, diversity, or composition of gut microbial communities associated with progeny fish from fishmeal or soybean meal fed broodstock. Hence, the mechanism behind nutritional programming does not seem to be associated with modified gut microbiome.

**Abstract:**

Nutritional programming (NP) is a concept in which early nutritional events alter the physiology of an animal and its response to different dietary regimes later in life. The objective of this study was to determine if NP via broodstock with dietary plant protein (PP) has any effect on the gut microbiome of the progeny fish and whether this modified gut microbiome leads to better utilization of PP diet. The experiment consisted of four different treatments as follows: (1) progeny that received FM diet obtained from fishmeal (FM)-fed broodstock (FMBS-FM, +control); (2) progeny that received PP diet obtained from FM-fed parents (FMBS-PP); (3) progeny that received PP diet obtained from “nutritionally programmed” parents (PPBS-PP; −control); and (4) progeny that received FM diet obtained from “nutritionally programmed” parents (PPBS-FM). Zebrafish was used as a model species. This study found that parental programming seems to have some positive effect on dietary PP utilization in progeny. However, the influence of NP with PP through broodstock on gut microbiota of the offspring fish was not detected.

## 1. Introduction

The cost of increasing fish production prices derived from increase in cost of ingredients of marine origin poses a significant challenge to the industry and threatens access to high quality protein in regions where food security is fragile to slight changes in market prices [1]. Although the inclusion rates of fishmeal in compound feeds for aquaculture have shown a clear downward trend, more recent estimates by FAO [2] predict that fishmeal prices, alone, may increase 30% by 2030 suggesting the need to continuously search for more cost-effective alternatives. However, the issues with using alternative protein sources, such as soybean meal (SBM) as a replacement of fishmeal (FM) in diets are still evident and include low palatability, essential nutrient deficiencies, impaired fish growth, intestinal inflammation, and gut microbiome dysbiosis [3,4,5,6]. One of the most recent approaches in mitigating the negative effects of dietary plant protein (PP) has been nutritional programming (NP). Nutritional programming is the concept that a brief early exposure to a dietary component, such as SBM, will ‘program’ an animal to better utilize the same dietary component when encountered later in life. The mechanism behind NP remains vague but it is believed to be induced through epigenetic changes, which are heritable changes that are not due to the actual alterations in DNA sequence [7,8,9]. Examples of these changes include methylation of cytosines in regulatory elements of genes and histone modification, both of which can alter gene expression. These marks can last the whole life of the animal, change its phenotype, and persist through generations [8]. However, the question concerning the genes which are affected by NP remains unclear [10,11].

Previous studies have achieved positive results using NP in both omnivorous and carnivorous fish species [5,11,12,13,14,15] but most focus on early fish stages and little is known about the importance of NP through broodstock and its impact on the well-being of progeny including its gut microbiome. Izquierdo et al. [15] was one of the first groups that studied NP through broodstock and observed improved growth of four-month-old gilthead seabream (*Sparus aurata*) juveniles fed low FM and low fish oil diets by previously exposing the broodstock of those fish to high vegetable-based feeds. Those authors later also showed that the same fish at 16-month of age were still able to grow on low FM/fish oil diets better compared to control group suggesting positive long-term effect of NP on utilization of vegetable-based diet [16].

While many different aspects influence the gut microbiome of fishes, environmental factors, feeding habits, and genetics are considered to be the most influential [17]. The composition of gut microbial communities also significantly changes as fish age, specifically with regards to intestinal morphology [18]. For example, Patula et al. [13] found that zebrafish (*Danio rerio*) gut microbiome showed differences throughout the fishes life no matter what feed was provided, presenting a natural progression of the zebrafish gut microbial structure with age. Since host genetics are a strong determinant of the intestinal microbiota of larval fish [19,20], our hypothesis was that a brief exposure of adult (broodstock) fish to a PP diet would cause an epigenetic change that would be transferred to the progeny and affect the intestinal environment of the offspring fish, specifically their gut microbiome, allowing them to utilize PP diet more efficiently. Therefore, the objective of this study was to determine if NP via zebrafish broodstock with dietary PP has any effect on the gut microbiome of the progeny fish and whether this modified gut microbiome leads to better utilization of PP diet. Zebrafish displays middle-ground tolerance to SBM with inflammation occurring at inclusions beyond 50% [21] suggesting its significant use in studies on diet-induced inflammation in the intestines of aquaculture species [22]. Furthermore, zebrafish can feed on vegetal and animal protein sources, and therefore the species has been suggested as a model to study the nutritional pathways and host-microbiome interactions in both carnivorous and omnivorous aquaculture species [23,24].

## 2. Materials and Methods

The study was conducted in the Center for Fisheries, Aquaculture, and Aquatic Sciences at Southern Illinois University, Carbondale (SIUC). All experiments were carried out in accordance with the recommendations in the Guide for the Care and Use of Laboratory Animals of SIUC. The SIUC Institutional Animal Care and Use approved all of the protocols performed (protocol# 18-007). During fish handling anesthesia was performed using water bath immersion in Tricaine methanesulfonate (MS-222) (Syndel, Ferndale, WA, USA) at a concentration of 0.01 mg/mL, a concentration far less than the suggested dose for euthanasia [25]. All efforts were made to minimize pain, stress, and discomfort in the animals. In addition, efforts were made to minimize the number of fish used where possible, and ensure as much data was obtained as possible from each fish used in the study. All welfare assessments were conducted on a daily basis from which welfare concerns were recorded and the fish cared for accordingly.

The zebrafish experiment was conducted in a zebrafish-rearing system (Pentair Aquatic Eco-systems, Cary, NC, USA). The recirculated system was equipped with two mechanical filters, a carbon filter, a biofilter, and a UV light. The flow rates of 300 mL min^−1^ were used during the experiment and 15% automatic daily water exchange of system volume was used and only 1/3rd of system capacity was utilized, which allowed for efficient waste removal and avoided accumulation of metabolites in the system. The biofilter was also cycled to guarantee proper ammonia reduction to NO_3_^−^ before the start of the trial. The average water temperature during the trial period was 26.9 ± 1.7 °C, while the average pH and conductivity were 7.12 ± 0.52 (GF Signet 3-2724, Irwindale, CA, USA) and 1012 ± 24 µS cm^−1^ (GF Signet 3-2850, Irwindale, CA, USA), respectively. The photoperiod consisted of 14 h of darkness and 10 h of light. The illumination was set at 245 lux (LX105 Lux Meter, Lutron Electronics Company, Coopersburg, PA, USA) and the distance between the water surface and the light source was ~10 cm.

### 2.1. Broodstock Rearing and Spawning

Zebrafish were SIUC domesticated individuals that originated from broodstock stocked to SIUC system in 2018. The original source of Zebrafish were obtained from a local pet store (Petco, Carbondale, IL, USA). Broodstock fish were fed initially with a commercial diet (Otohime C2, Tokyo, Japan). Two weeks before the experiment began, the broodstock fish were spawned to release any sperm or eggs in order for all of the fish to share similar baseline in terms of gonad maturation stage. The broodstock were then moved to separate treatment groups and fed two to three times a day *ad libitum* with FM-based or SBM-based diet. The FM and SBM broodstocks were kept in separate 10 L tanks. The feed intake of both FM and SBM diets was closely monitored to ensure high feed intake. Both groups were also fed *ad libitum* with *Artemia* nauplii (given after feeding with FM or SBM diets to ensure consumption of the dry feed) to provide additional nutritional supplementation for proper gonad development. Since adult zebrafish are characterized by approximately four-week gametogenesis cycle [26], the experimental FM and SBM diets were introduced during a two-week period of exposure that was considered long enough to ensure that the potential epigenetic change caused by SBM in the broodstock would be passed on to the developing gametes, but short enough to prevent any side-effects that could be caused by presence of isoflavonoid phytoestrogens—phenolic compounds found in SBM known to induce estrogenic properties by binding to estrogen receptors, which can impair spawn and gamete quality [27]. These two weeks of feeding were considered the ‘programming stage’ of the SBM broodstock.

When it was time to breed, there was a 1:1 ratio of females to males, 10 females and 10 males [28]. A plastic mesh of 4 mm was placed in a breeding tank (10 L) with a fake plant to induce spawning. The fish were left to breed for 24 h and then the broodstock were removed. The wire mesh was taken out, and eggs hatched at 27 °C. At three days post hatch (dph) when the majority of the larvae were actively swimming, they were randomly distributed into experimental tanks (3 L) at a density of 40 larvae per tank for a total of 12 tanks with 3 replicates per treatment.

Larval zebrafish were fed with saltwater rotifers (*Brachionus plicatilis*) starting from 3–7 dph. The rotifers were obtained from cysts purchased from a commercial vendor (Brine shrimp direct, Ogden, UT, USA). The rotifers were kept in a 30 L bucket. The salinity was held at 15 parts per thousand (ppt), the temperature was kept at 23–25 °C, and constant aeration and light were supplied to the bucket. The rotifers were fed twice a day with powdered blended *Spirulina* sp. (Earthrise Nutritionals, Irvine, CA, USA). The rotifer culture water was changed daily (min. 50–70%). 

Zebrafish were then fed with *Artemia* nauplii together with rotifers from 7–10 dph and then *Artemia* only from 10–12 dph. To induce *Artemia* hatching, *Artemia* cysts (GSL brine shrimp, Ogden, UT, USA) were added to Macdonald jars and incubated for 24 h under constant light and aeration at 25 °C and salinity of 30 ppt. After 24 h, hatched *Artemia* nauplii were harvested through a 150 µm sieve, rinsed under freshwater, and scooped into the tanks for feeding.

### 2.2. Diet Preparation

All of the experimental feeds were formulated and produced at SIUC. Two different types of diets were made: SBM or FM-based (Table 1). The SBM diet served as the PP treatment. The dietary formulations used in experimental diets in the present study followed formulations from our previous studies investigating the effects of dietary soybean meal on zebrafish, which have been recently published [6,7,8,29]. The level of protein and lipid included in diets followed dietary protein and lipid levels in commercial formulations for zebrafish (Otohime, Japan; Zeigler, PA, USA; Skretting, Norway). Furthermore, the levels of dietary protein in the feed formulations were assumed to be sufficient to meet the requirements of all essential amino acids and therefore maximum weight gain [30]. All other nutrients were included to meet requirements of zebrafish following recommendations from NRC [31] for cyprinids.

The dry protein ingredients (FM, krill meal, and SBM) were first added to a centrifugal mill, (Zm 100, Retsch Haan, Germany) and ground to 500 µm. After the centrifugal mill, all ingredients were manually sieved through a 255 µm sieve to ensure all particles were of the appropriate and uniform size. All of the dry ingredients (excluding soy lecithin and choline chloride) were added together and mixed for 15 min (KitchenAid Countertop Appliances, Benton Harbor, MI, USA). After all of the dry ingredients were mixed, the fish oil was added with the soy lecithin dissolved in the oil to ensure even amounts of lecithin throughout the feed. The oil and dry ingredients were mixed again for 15 min. After the oil and dry ingredients were finished mixing, water with dissolved choline chloride (15% of total mass of feed) was added to ensure even mixing. Next, the feeds were processed using an extruder (Caleva Extruder 20, Sturminster Newton Dorset, England) to produce “noodles”. Feed was slowly added to the extruder at levels between 20–24 revolutions per minute (rpm) to obtain a proper noodle size. After the noodles were made, they were processed using a spheronizer (Caleva, Sturminster Newton Dorset, England) at 600 rpm for 3 min, 1800 rpm for 30 s, and then 600 rpm for 2–5 min to finish the process. The noodles were added to the spheronizer to obtain proper size of uniform spheres for feeding and to ensure high nutrient retention. Finally, the pellets were freeze-dried (Labconco, Kansas City, MO, USA). After drying pellets were sieved to appropriate size using a vibratory sieve shaker (Retsch Hann, Germany). The shaker assorted the pellets ending at a powder form (<155 µm) and starting at the biggest pellet size (>800 µm) with several sizes in between. All finished feeds were stored in bags in −20 °C to avoid oxidation. While the feeds were used in experimentation, they were kept at 4 °C.

Proximate composition of diets included quantification of the following: crude protein, crude lipid, moisture, and ash (Table 1). Briefly, samples were analyzed for ash by combustion (550 °C for 5 h) in a muffle furnace (Lindberg Blue M, MA, USA); crude protein (N × 6.25) using a Leco nitrogen analyser (Model FP-628, Leco Corporation, St. Joseph, MO, USA); and crude lipid was extracted with chloroform–methanol (2:1, *v*/*v*). All diets were analyzed in triplicates.

### 2.3. Experimental Groups and Feeding Regime

**Figure 1:**
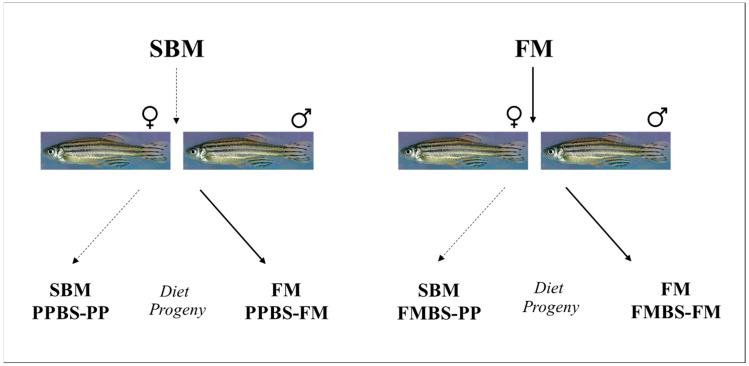
The experimental design of the study. Broodstock fish were fed soybean meal (SBM) or fishmeal (FM)-based diets for two weeks and the offspring from each group was then challenged with SBM diet.

The feeding trial consisted of four different treatments as follows:

1. A progeny obtained from FM-fed broodstock that received FM diet (FMBS-FM, +control);
2. A progeny obtained from FM-fed broodstock that received PP diet (FMBS-PP, −control);
3. A progeny obtained from “nutritionally programmed” broodstock that received PP diet (PPBS-PP);
4. A progeny obtained from “nutritionally programmed” broodstock that received FM diet (PPBS-FM) (Figure 1).

Fish were fed *ad libitum* from 3–22 dph to ensure high feed intake. From 22–48 dph, all groups of fish were fed with a restricted daily feeding rate during the feeding trial. Each meal was observed to ensure that all feed added to the tank was consumed by the fish. In addition, the feeding rate was adjusted daily, using an assumed FCR of 1, and also readjusted through observations of feed intake at each feeding. Also, a weekly weighing was conducted during the restricted feeding period in order to determine the actual biomass in each tank and, readjust the feeding rate accordingly. The feeding rate was originally set by measuring the observed feed intake for each tank and setting the feeding level to the tank with the lowest feed intake. This ensured a consistent feeding rate across all tanks and ensured all food added to the tanks was consumed. Most importantly, this eliminated any potential bias that would have been caused by palatability or preference differences between FM and SBM diets that could have influenced the feed intake among groups. This helped ensure that any differences detected in growth metrics would not be a function of different feed consumption levels. The restricted feeding also allows for better dietary nutrient utilization compared to *ad libitum* feeding [32] which was critical (especially with respect to the SBM feed).

### 2.4. Sampling and Measurements

The measured responses that were assessed included: average weight (measured at 20, 27, 34, 41, and 48 dph), weight gain for each treatment group, and gut microbiome diversity and composition. At each weighing, netted fish were patted dry on a paper towel prior to being placed on a tared analytical scale. The average weight gain was calculated per treatment starting the day of the first progeny weighing till the end of the study. The average weight gain was calculated as follows:

Final mass (g) − initial mass (g) = Weight gain (g)

[(Final mass (g) − initial mass (g))/initial mass (g)] × 100 = Weight gain (%)

Samples were taken throughout the study to assess the structure of the microbiome for each treatment at the following stages: egg, larva, and juvenile. Egg samples were taken after spawning from each parental group (FM and SBM), and placed into Eppendorf tubes (enough to fill up 2 mL volume). Similarly, freshly hatched larvae were sampled from each parental group (FM and SBM) into Eppendorf tubes (enough to fill up 2 mL volume). The last sampling was conducted when the progeny fish were 48 dph at the end of the feeding trial and three fish from each replicate tank were sampled. All samples were frozen directly in liquid nitrogen to preserve the gut microbiome and stored at −80 °C until analyzed. 

### 2.5. DNA Extraction and 16S rRNA Gene Sequencing 

The exterior surface of juvenile fish was swabbed with 70% ethanol before dissection of the whole intestine using sterile instruments. Larval fish and eggs were washed in 70% ethanol for one minute and then washed for 30 s under distilled water to remove any attached bacteria. DNA was isolated from intestines (juvenile fish) or whole samples (larval fish and eggs) using a MO BIO PowerSoil isolation kit (QIAGEN, Hilden, Germany). Following isolation, DNA was quantified using a Qubit 3.0 fluorometer (ThermoScientific, Waltham, MA, USA).

Amplicon library preparation and sequencing was performed at the John G. Shedd Aquarium’s Molecular and Microbial Ecology Lab (Chicago, IL, USA). Bacterial and Archaeal DNA was amplified using primer constructs (515f/806rB) targeting the V4 region of the 16S rRNA gene [33]. The constructs contain Illumina specific adapters followed by 12 bp Golay barcodes on each forward primer, primer pads and linkers as well as the template specific PCR primer at the 3′ end. Mock microbial community DNA standards (Zymo Resarch) and negative controls containing no template DNA were included as PCR controls. Thermal cycling conditions were carried out as follows: 98 °C for 30 s, 30 cycles at 98 °C for 10 s, 55 °C for 30 s and 72 °C for 30 s, with a final extension of 5 min at 72 °C. Gel electrophoresis was used to visually check for successful amplification. Amplicons were then cleaned and normalized using the SequalPrep™ Normalization Plate Kit (Applied Biosystems, Waltham, MA, USA), and pooled in equal volumes. The pooled amplicon library was quantified using a Qubit 3.0 fluorometer and Qubit dsDNA HS Assay Kit, then further cleaned and concentrated using the UltraClean PCR Clean-Up Kit (QIAGEN). The pooled library was diluted to 2 nM before denaturation and further dilution to a loading concentration of 6 pM. Paired-end sequencing for a total of five hundred cycles was conducted on an Illumina MiSeq platform using custom sequencing primers described previously [34] with the addition of 10% PhiX Control library (Illumina, San Diego, CA, USA) to increase sequence diversity.

### 2.6. Bioinformatic Analysis

Raw sequence reads were processed using a combination of QIIME2 version 2018.11 [35] and phyloseq [36]. Demultiplexed reads were imported into QIIME2 and denoised with DADA2 [37], and reads were trimmed at 13 bp and truncated after 230 bp. DADA2 filters sequences for quality, removes chimeric sequences, and produces amplicon sequence variants (ASVs). A rooted phylogenetic tree was then generated using the ‘q2-phylogeny’ pipeline under default settings, and taxonomy was assigned to ASVs using a Naïve Bayes classifier trained on the SILVA release 132 99% OTUs database [38], where sequences had been trimmed to include only the bases within the V4 region bound by the 515F/806R primer pair. Reads that mapped to chloroplast and mitochondrial sequences were filtered from the sequence variants table using the ‘filter_taxa’ function. Data was then imported into phyloseq using the “import_biom” and ‘import_qiime_sample_data’ functions and merged into a phyloseq object. Richness (observed ASVs) and Faith’s phylogenetic distance (FPD) were calculated for all samples with phyloseq and the ‘estimate_pd’ function from the btools package. ASV counts were then normalized using cumulative sum scaling [39] and beta-diversity was analyzed using unweighted, generalized, and weighted UniFrac distances calculated using phyloseq and the GUniFrac package. From these distances, non-metric multidimensional scaling (NMDS) was performed and plotted, and a permutational multivariate analysis of variance (PERMANOVA) was used to test for significant differences in community structure using the vegan [40] and pairwise Adonis [41] packages. To ensure significant differences were not the result of unequal dispersion of variability between groups, permutational analyses of dispersion (PERMDISP) were conducted for all significant PERMANOVA outcomes using the vegan package. Hierarchal clustering was performed using Ward’s agglomeration clustering method [42] on generalized UniFrac distances and the ‘hclust’ function. Further, the relative abundances of ASVs within each sample were calculated and plotted using phyloseq.

### 2.7. Statistical Analysis

Unless specified otherwise, R version 3.6.3 [43] was used for statistical analyses. Pairwise Wilcoxon rank-sum tests were performed using a Benjamini-Hochberg correction for multiple comparisons for comparing alpha diversity and relative abundances. Differences in beta diversity were tested using a pairwise PERMANOVA with a Benjamin-Hochberg correction and 9999 permutations. Pairwise PERMDISPs were carried out for all significant PERMANOVAS using 9999 permutations to test for significant differences in the variability of dispersions. For the final average weight and weight gain data, two-way ANOVA was run, followed by a Tukey’s post-hoc test with a significance set at *p* < 0.05 using SPSS (Chicago, IL, USA, version 25).

## 3. Results

### 3.1. Growth Performance and Survival

Table 2 presents survival of the fish throughout the experiment. No statistical differences were detected in the survival among the groups. Table 3 presents both the final weight gain in grams and percent, and final average weight of fish at the end of the feeding trial. The two-way ANOVA detected a significance for the overall test (n = 3, 6, 12; *p* < 0.05), but individual treatments and parents did not present any statistical effect (two-way ANOVA, n = 3, 6, 12; *p* = 0.001). The PPBS-PP group achieved similar weight gain as the (+) control and the PPBS-FM groups in terms of grams. Although PPBS-PP group achieved weight gains that were numerically higher than the (−) control, significance was not detected. The final average weight results presented similar trend as weight gain in grams. When weight gain was converted into percent, there was no significance detected between any of the groups.

### 3.2. Microbial Richness and Diversity

**Figure 2:**
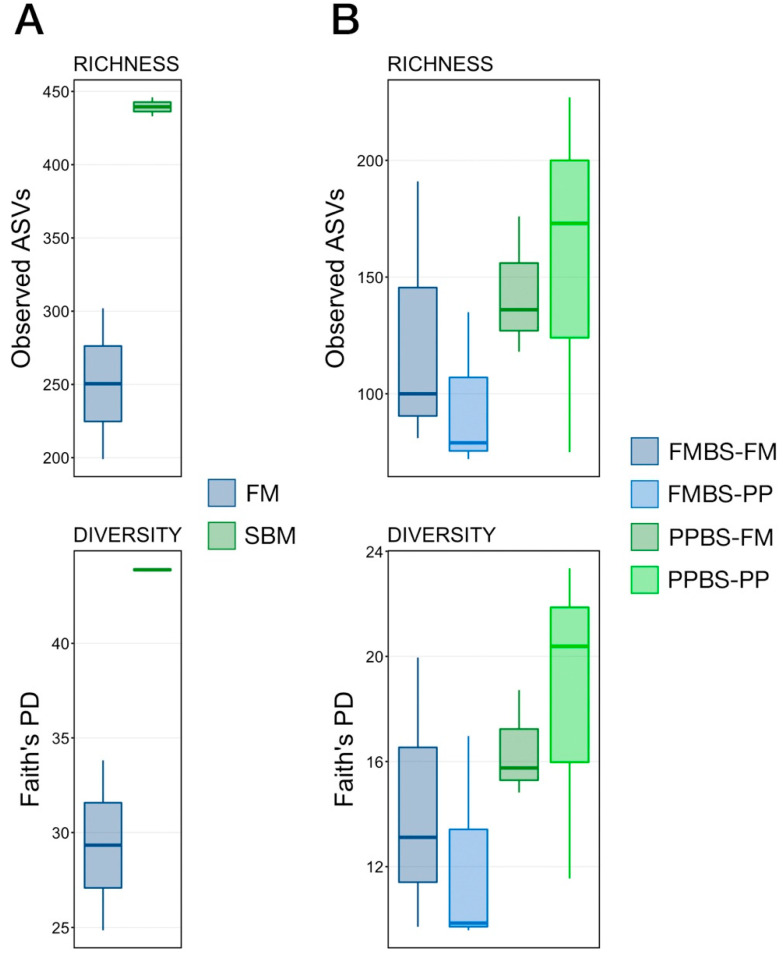
Boxplots demonstrating the richness (Observed ASVs) or diversity (Faith’s phylogenetic distance) of larval fish at 3 dph (**A**) or progeny adult fish at 48 dph (**B**). There were no statistical differences detected. Abbreviations: dph, days post hatch; FM, fishmeal broodstock; SBM, soybean meal broodstock; FMBS-FM, fishmeal broodstock with fishmeal programming; FMBS-PP, fishmeal broodstock with soybean meal programming; PPBS-RM, soybean mean broodstock with fishmeal programming; PPBS-PP, soybean meal broodstock with soybean meal programming.

Microbial richness (observed ASVs) and diversity (Faith’s phylogenetic diversity) were compared between progeny groups at the larval stage (3 dph) and at the final timepoint of the offspring feeding trial (48 dph). While the richness and diversity of larval fish from PP broodstock were higher than those from FM broodstock, those differences were not statistically significant (Figure 2A; Kruskal-Wallis, n = 2, *p* > 0.05). Similarly, richness and diversity were higher at 48 dph in the offspring of PP broodstock but owing to small sample size the differences were not significant (Figure 2B; pairwise Wilcoxon rank-sum test with Benjamini-Hochberg correction, n = 3, *p* > 0.05).

### 3.3. Microbial Community Composition

**Figure 3:**
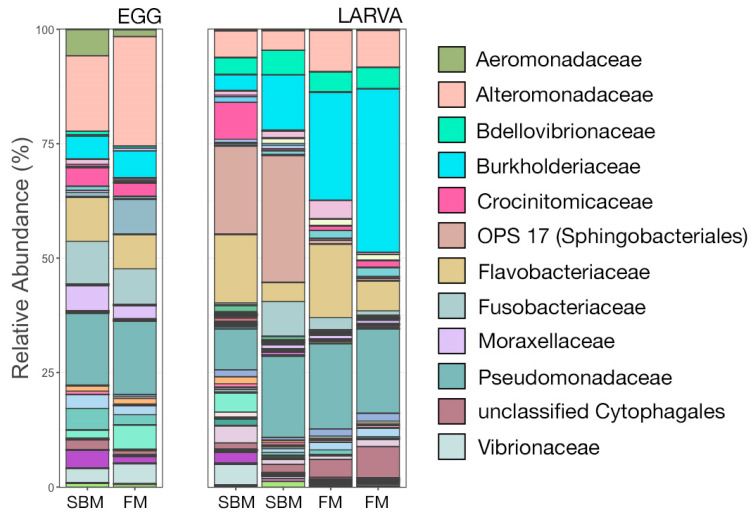
Bar plots showing the relative abundance of microbial families within egg samples and from larval samples (3 dph) obtained from spawning SBM-fed or FM-fed broodstock. Abundances were normalized to the total number of ASVs within each sample. The twelve most abundant families across all samples are displayed in the legend. Abbreviations: dph, days post hatch; FM, fishmeal; SBM, soybean meal.

The composition of microbial communities associated with eggs and larval fish were visualized at the taxonomic rank of family. Eggs from PP and FM broodstock had similar microbial communities, with the most abundant families being Altermonadaceae, Pseudomonadaceae, Fusobacteriaceae, and Flavobacteriaceae for both groups (Figure 3). Interestingly, Aeromonadaceae was in higher relative abundance in eggs from FM broodstock. However, due to a single replicate, no statistical comparisons between community structure were made for egg-associated communities. There were more noticeable differences in the composition of larval fish-associated communities, with OPS 17 (an environmental family within Sphingobacteriales) being very abundant in larva from PP broodstock, yet virtually absent in FM larva (Figure 3). Conversely, members of Burkholderia were considerably more abundant in communities in larva from FM broodstock. Members of Alteromonadaceae, Bdellovibrionaceae, Flavobacteriaceae, and Pseudomonadaceae were among the most abundant in both groups of larval fish (Figure 3). Again, due to small sample size (n = 2), statistical comparisons of community composition were not made for larva and should be interpreted with caution.

**Figure 4:**
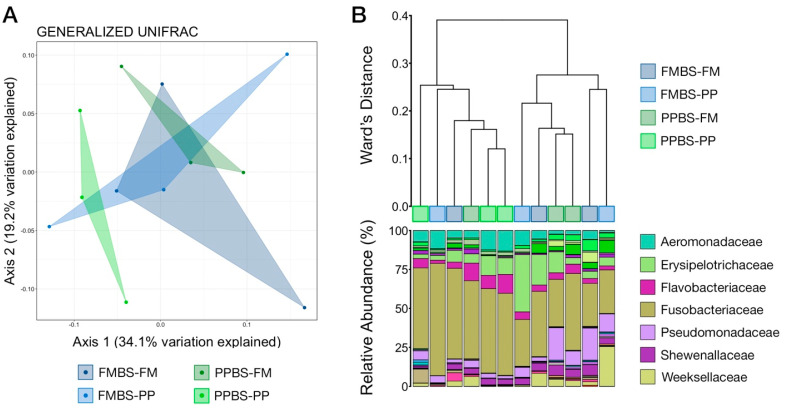
(**A**) Principal coordinates analysis (PCoA) of unweighted Unifrac distances relating the variation in microbial community composition between samples from the final weights time point (48 dph). There was no significant difference detected in community composition between groups (PERMANOVA, n = 3, *p* > 0.05). (**B**) Hierarchal clustering showing the relatedness of gut microbial communities from 48 dph based on generalized UniFrac distances. The barplot demonstrates the relative abundance of microbial families and the seven most abundant families are displayed in the legend. Abbreviations: dph, days post hatch; FM, fishmeal broodstock; SBM, soybean meal broodstock; FMBS-FM, fishmeal broodstock with fishmeal programming; FMBS-PP, fishmeal broodstock with soybean meal programming; PPBS-RM, soybean mean broodstock with fishmeal programming; PPBS-PP, soybean meal broodstock with soybean meal programming.

Differences in microbial community structure between the progeny groups (i.e., FMBS-FM, FMBS-PP, PPBS-FM, and PPBS-PP) at the final sampling point (48 dph) were analyzed using NMDS, hierarchal clustering, and PERMANOVA. There were no significant differences in the composition of gut microbial communities from the four progeny groups at 48 dph (Figure 4A; pairwise PERMANOVA, n = 3, *p* > 0.05). Visualization with NMDS illustrated that the microbial communities from each treatment group were not distinct from the others and did not cluster based on broodstock diet or nutritional-programing group (Figure 4A). Hierarchal clustering based on generalized UniFrac values revealed that gut communities from treatment groups were no more similar to communities within the same treatment group than they were to other treatments (Figure 4B). Further, both main clades of six communities contained intermixed treatment groups, suggesting that differences in community structure were not explained by treatment group (i.e., broodstock diet and nutritional programming). Visualization of community structure at the taxonomic rank of family demonstrated that the driving force between the two most distinct clades was likely the differential abundance of Fusobacteriaceae (more abundant in the left clade), and Pseudomonadaceae and Weeksellaceae (both more abundant in the right clade), which were among the most abundant families (Figure 4B). Across all gut communities at 48 dph members of Fusobacteriaceae were the most predominant, followed by Erysipelotrichaceae, Pseudomonadaceae, Aeromonadaceae, and Flavobacteriaceae (Figure 4B).

**Figure 5:**
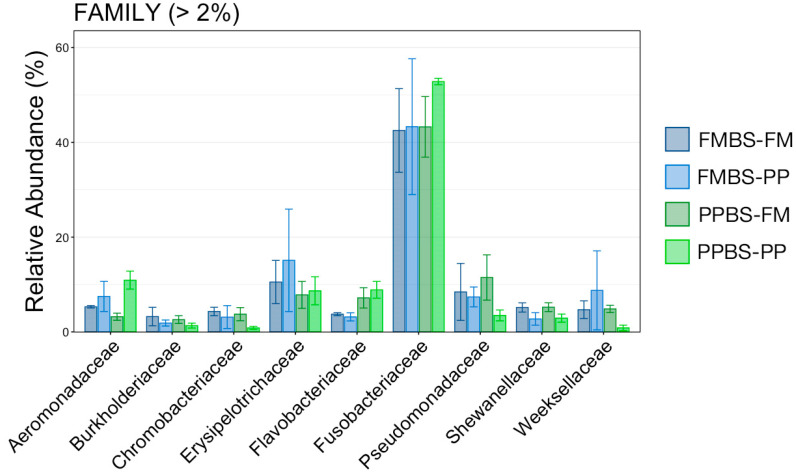
Barplot showing the relative abundance of the nine most abundant taxonomic families within gut microbial communities at 48 dph. Error bars demonstrate the standard error of the mean of the total relative abundance of each family from each treatment group. There were no significant differences (pairwise Wilcoxon rank-sum test, n = 3, *p* > 0.05). Abbreviations: dph, days post hatch; FM, fishmeal; SBM, soybean meal; FMBS-FM, progeny fish fed FM diet that originated from FM-fed broodstock; FMBS-PP, progeny fish fed PP diet that originated from FM-fed broodstock; PPBS-FM, progeny fish fed FM diet that originated from PP-fed broodstock; PPBS-PP, progeny fish fed PP diet that originated from PP-fed broodstock.

To further characterize potential differences between treatment groups at 48 dph, the relative abundances of every taxonomic family comprising more than 2% of the overall microbial community were directly compared (Figure 5). Fusobacteriaceae, the most abundant family (>40% of overall microbial community in all groups), was in higher relative abundance within gut communities from PPBS-PP progeny but owing to small sample sizes and large inter-sample variation the increase was not significant (Figure 5; pairwise Wilcoxon rank-sum, n = 3, *p* > 0.05). Conversely, Chromobacteriaceae and Weeksellaceae were less abundant within PPBS-PP progeny gut communities than all other groups, but once again the difference was not significant (Figure 5; pairwise Wilcoxon rank-sum, n = 3, *p* > 0.05). Flavobacteriaceae was more abundant in both PP and FM-fed progeny that came from PP broodstock versus FM broodstock, but the difference was not significant. The remaining five families whose members represented more than 2% of the overall community (Aeromonadaceae, Burkholderiaceae, Erysipelotrichaceae, Pseudomonadaceae, and Shewanellaceae) were largely similar across treatments (Figure 5). 

## 4. Discussion

### 4.1. Growth Performance

The PPBS-PP group were progeny fish that originated from SBM-fed parents and were given a SBM diet throughout the progeny stage trial. Although there was no statistical difference detected, at the end of the progeny feeding trial the PPBS-PP group had numerically higher weight gain and average weight compared to the FMBS-PP group (progeny obtained from FM-fed broodstock that received SBM diet throughout the duration of the study). Furthermore, the weight gain in terms of grams and average weight of the PPBS-PP group were not statistically different compared to the two groups that were fed a FM diet (PPBS-FM and FMBS-FM). Conversely, the weight gain and average weight of FMBS-PP were significantly lower compared to both FMBS-FM and PPBS-FM groups. These results could possibly suggest some positive parental effect that allowed the fish in the PPBS-PP group to perform on PP diet as well as those fish that received feed based on fishmeal. Although the large variations observed in the growth data between fish within tanks and within groups make the conclusions largely speculative, the data seem to support evidence from other studies indicating that phenotypic alterations induced by epigenetic changes derived from environmental programming might not be evident at first. Yellow perch (*Perca flavescens*) that were programmed with SBM had numerically higher weight gains during a ‘PP challenge’ compared to the non-programmed group but a significant difference was not observed [14]. Moreover, rainbow trout (*Oncorhynchus mykiss*) broodstock programmed with three different diets varying in FM levels (64, 34, and 0%) produced progeny that did not present any maternal effects from programming in the beginning, before the progeny were exposed to (challenged with) the ‘maternal diets’, but maternal effects appeared later—three weeks into the study [44]. However, the impacts of broodstock nutrition on the well-being of the offspring are evident. For example, when rainbow troutbroodstock were fed either a methionine deficient diet (0%, or 0.5%), or a control—methionine containing diet (1.5%), the trout alevins from the methionine deficient group exhibited down regulation of important growth factors [45].

A high inclusion of dietary PP can lead to reduced growth in fish [46,47,48] Previous studies revealed that as higher levels of soy protein isolate were used to substitute FM in diets at varying replacement levels (0, 25, 50, 75, 87.5, and 100%), specific growth rate decreased in fish fed formulations containing 50–100% of the PP [49]. It is also well-known that isoflavonoid phytoestrogens—phenolic compounds found in SBM known to induce estrogenic properties by binding to estrogen receptors, can impair spawn and gamete quality [27]. Sink et al. [50] fed channel catfish (*Ictalurus puncatatus*) broodstock for 69 days with diets differing in the level and source of protein and lipid (animal versus plant) and found that broodstock fish that received a PP diet (60% SBM-based) supplemented with fish oil suffered in spawn quality and quantity. Similarly, African catfish (*Clarias gariepinus*) broodstock fed a SBM diet compared to a FM diet for 84 days suffered in proper ovary and testis formation [51]. Furthermore, 20-week feeding of goldfish (*Carassius auratus*) with SBM-based diet led to disruption in sex hormone biosynthesis, reduced oocyte and spermatocyte maturation ultimately leading to low hatching rates [27]. The effects of dietary isoflavones on fish reproductive performance, however, can vary depending on exposure time and the isoflavone concentration [52]. Kemski et al. [14] also reported that yellow perch that had transitioned back to FM-based diet during gametogenesis after seven-month SBM diet feeding presented no differences in reproductive capacity. This possibly suggests that a sufficient level of recovery is possible after long-term exposure to high phytoestrogen level feeding. Interestingly, the zebrafish progeny originating from both broodstock groups (FM and PP) that received FM diet presented the same growth performance (in terms of weight gain and average weight). Although the effect of NP on gonad quality was not the focus of our study, the short (two-week) exposure to SBM diet did not seem to impair the gonadal formation and/or egg development since both broodstock groups produced viable offspring that was able to perform equally well on FM diet.

### 4.2. Gut Microbial Communities

The similarity of alpha diversity metrics (i.e., richness and phylogenetic diversity) in larval and juvenile fish from different broodstock groups might relate to their rearing environment, since all of the fish were occupying the same recirculated system where all abiotic conditions (temperature, pH, salinity, and conductivity) were equal in each tank. Further, the lack of replicates which resulted from insufficient egg and larva counts for sampling hindered statistical comparisons. The majority of the zebrafish larvae obtained from the spawning of each broodstock group were used in the feeding trial to ensure representative fish densities in all treatments. For that reason, insufficient egg and larvae samples were obtained for the microbiome analysis. Therefore, the limited microbiome data presented for both developmental stages should be interpreted with caution. Previous work has demonstrated that fish (aged 0–21 dph) living in the same environment have similar richness and diversity [53]. The larval fish in this study were at the stage of mouth opening, meaning they had not received their first feed yet, and the similarities between larva from different broodstock groups suggests that the external environment may have a larger impact on the developing microbiome. The importance of environmental factors have been demonstrated previously; Common carp (*Cyprinus carpio*), Silver carp (*Hypophthalmichthys molitrix*), and Bighead carp (*Hypophthalmichthys nobilis*) from a shared environment had similar gut microbial richness and diversity despite differences in their feeding habits and dietary sources [54].

In addition to similar richness and diversity, the composition of gut microbial communities in progeny fish from different broodstock groups subjected to differing feeding regimes were not significantly different. Although larval zebrafish can acquire some of their microbial makeup from their parents, fish seem to gain a large portion of their gut microbiome during the egg and early larval stages from other sources. These include microbes in the surrounding water, feed once mouths are open, habitat, and several abiotic factors that might affect the bacteria present such as temperature, salinity, and more [55]. Here, we showed that, with the exception of a couple microbial taxa, egg- and larva-associated communities were similar, suggesting that environmental factors likely shape their microbial makeup.

Diet has also been shown to impact the gut microbiota in fish, including reduction in numbers of beneficial microbes, and promotion of growth of deleterious bacteria in the gut. For example, it has been observed that complete replacement of FM with lower quality PP such as pea and soybean meals had negative effects on rainbow trout gut microbiota compared to trout fed a FM-based diet [56]. However, there have been other claims indicating that the diet alone does not necessarily dictate or have a major impact on the gut microbiome [57,58]. Japanese sea bass (*Lateolabrax japonicus*) fed diets containing 21, 35, or 42% SBM used as FM replacement presented no change in community composition in the gut among the treatments with Proteobacteria consisting of the majority of the fish gut microbiome [58]. Here, our results did not demonstrate much of an effect of diet or nutritional programming. Similarly, previous studies reported that rainbow trout fed a diet with 5% microalgae supplementation compared to a control diet containing 100% fish oil, presented no significant difference in the overall structure of the gut microbiome between treatments [57].

It has been well documented in humans that the passage of gut microbiomes from mother to child occurs during womb development and through the birthing process [59]. There is some evidence that fish can also ‘pass down’ gut microbiomes to their offspring. Research showed that the bacteria *Flavobacterium psychrophilum* was able to be transferred from the mother to the egg in sockeye salmon (*Oncorhynchus nerka*) where they were most likely transmitted from the ovarian fluid of the female to the surface of the egg [60]. Furthermore, the gut microbiome of fish is not necessarily passed down as it is in mammals but it is determined by selective pressures and host genotype [61]. For example, when a zebrafish gut microbiome was inserted into a gnobiotic mouse, the mouse gut selected for phyla that were more consistent with a ‘standard’ mouse gut microbiome, implying that animals have a ‘selective gut’ in terms of microbes occupying it [62]. A colonization by commensal microbes has also been found to be crucial in proper fish development. In newly hatched zebrafish, these microbes increase the resistance of the fish to viral infections through upregulation of several genes encoding proinflammatory and antiviral mediators. Interestingly, however, the induction of those genes which occurs through covalent modification of histone H3 at gene promoters is not directly altered by those commensal microbes [63].

The dominant bacterial families found within the guts of progeny fish were Fusobacteriaceae, Pseudomonadaceae, Erysipelotrichaceae, Aeromonadaceae, and Flavobacteriaceae. Indeed, these are considered a major component of the zebrafish gut microbiome [64] and typically considered a part of the core of gut microbiota in fish [65]. The similar gut microbiome development patterns and a similar ‘core microbiome’ present in omnivorous zebrafish and other carnivorous species [13,66] suggest zebrafish as a good model for potentially other fish species of commercial importance. There were no significant differences in the abundance of any of these dominant families between any of the treatment groups. Previous work using cod (*Gadus morhua*) has shown that as a fish ages the gut microbiome changes, but those changes are brought on by the developing intestinal environment, not the feed given [67]. It has also been noted that although the mother-progeny link is strong in higher vertebrates it is likely not as relevant in the fish gut microbiome [68]. Further, a study on Paddlefish (*Gadus morhua*) and Bighead carp showed that both species had differently structured gut microbiomes when raised in the same pond and given the same feed. The study also reported that within species, microbial communities were similar, showing that similar environments gave rise to similar gut communities [69]. The weak mother-progeny effect in fish, and the standardized environment between tanks, may explain why eggs from different treatments harbored the same microbes.

## 5. Conclusions

This study was the first attempt to investigate the effect of NP via broodstock with PP on the progeny of zebrafish including their gut microbiome. Although not significantly different a strong trend was observed showing better growth performance between progeny fish fed SBM diet that originated from broodstock exposed to SBM as opposed to progeny fish fed SBM diet that came from FM-fed broodstock. Contrary to other research, the two-week exposure of the zebrafish broodstock to SBM diet did not seem to have any negative effect on the viability of offspring fish. However, future studies should investigate the effects of NP with PP on gamete quality.

This study also found no changes in the richness, diversity, or composition of gut microbial communities associated with progeny fish from FM or PP-fed broodstock. Overall, it does not seem that NP via broodstock impacts the gut microbiota of the progeny fish. In addition, the evidence seems to indicate that the gut microbiome in zebrafish is possibly more influenced by the surrounding environment than the diet provided. Future studies should elucidate further the mechanism behind NP to allow for its better utilization as a tool to improve growth performance of fish fed PP-based feeds. Specifically, an assessment of epigenetic effects of NP on gene expression in form of an identification of specific methylation sites should be explored to further our understanding on the genesis of improved growth performance in programmed fish.

## Figures and Tables

**Table 1:** Feed formulation of experimental diets (g/100 g) and their proximate composition (g/100 g).

Ingredient	FM	SBM
Fish Meal ^1^	63.8	-
Soybean Meal ^2^	-	46.3
Soy Protein Isolate ^3^	-	15.4
Krill Meal ^4^	10.0	10.0
CPSP ^5^	5.8	5.7
Dextrin ^6^	5.3	-
Fish Oil ^7^	3.9	7.1
Soy Lecithin ^8^	4.7	4.7
Mineral Mix ^9^	2.4	2.4
CaHPO_4_^6^	-	1.4
Vitamin Mix ^10^	2.0	2.0
Vitamin C ^11^	0.1	0.1
Choline Chloride ^6^	0.1	0.1
Methionine ^6^	-	0.5
Lysine ^6^	-	2.3
Threonine ^6^	-	0.1
Taurine ^6^	0.9	0.9
Guar Gum ^6^	1.0	1.0
**Sum**	**100**	**100**
**Analyzed composition**		
Crude protein (N × 6.25)	54.51 ± 0.57	53.30 ± 0.13
Crude lipids	17.25 ± 0.47	16.89 ± 0.08
Ash	15.39 ± 0.09	9.10 ± 0.27

^1^ Mechanically extracted menhaden meal, stabilized with 0.06% ethoxyquin (Omega Protein, Reedville, VA, USA). ^2^ Solvent extracted soybean meal (Premium Feeds, Perryville, MO, USA). ^3^ Crude protein concentration min. 92% (Dyets Inc., Bethlehem, PA, USA). ^4^ Proccesed *Euphausia superba* (Florida Aqua Farms, Dade City, FL, USA). ^5^ Soluble fish protein hydrolysate (Sopropeche S.A., Boulogne Sur Mer, France). ^6^ Dyets (Bethlehem, PA, USA) ^7^ Cod liver oil (MP Biomedicals, Solon, OH, USA). ^8^ Refined soy lecithin (MP Biomedicals, Solon, OH, USA). ^9^ Bernhart-Tomarelli mineral mix with 5 ppm selenium in a form of sodium selenite (Dyets, Bethlehem, PA, USA). ^10^ Custom Vitamin Mixture (mg/kg diet) Thiamin HCl, 4.56; Riboflavin, 4.80; Pyridoxine HCl, 6.86; Niacin, 10.90; D-Calcium Pantothenate, 50.56; Folic Acid, 1.26; D-Biotin, 0.16; Vitamin B12 (0.1%), 20.00; Vitamin A Palmitate (500,000 IU/g), 9.66; Vitamin D3 (400,000 IU/g), 8.26; Vitamin E Acetate (500 IU/g), 132.00; Menadione Sodium Bisulfite, 2.36; Inositol, 500 (Dyets, Bethlehem, PA, USA). ^11^ L-Ascorbyl-2-Polyphosphate (Argent Aquaculture, Redmond, WA, USA).

**Table 2:** Survival (%) of zebrafish throughout the experiment.

Group	Week 1	Week 2	Week 3	Week 4	Final Weighing
FMBS-FM	100	92.3 (±8.4)	92.3 (±8.4)	79.0 (±11.0)	79.0 (±11.0)
FMBS-PP	100	99.2 (±1.3)	96.7 (±3.8)	83.1 (±3.0)	83.1 (±3.0)
PPBS-FM	100	100.0 (±0.0)	97.1 (±5.1)	82.6 (±5.3)	82.6 (±5.3)
PPBS-PP	100	95.9 (±3.6)	95.9 (±3.6)	80.7 (±4.1)	80.7 (±4.1)

**Table 3:** Growth performance data obtained at 48 dph (end of the progeny feeding). Different letters indicate a statistical difference between groups (*p* < 0.05).

Treatment	Weight Gain (g)	Weight Gain (%)	Average Weight (g)
FMBS-FM	0.157 ^a^ (±0.029)	1281.27 (±298.17)	0.170 ^a^ (±0.032)
FMBS-PP	0.090 ^b^ (±0.028)	745.84 (±459.84)	0.104 ^b^ (±0.025)
PPBS-FM	0.164 ^a^ (±0.010)	1289.81 (±20.54)	0.177 ^a^ (±0.011)
PPBS-PP	0.114 ^ab^ (±0.028)	852.50 (±452.53)	0.130 ^ab^ (±0.023)

## Data Availability

Data can be available upon direct request to the corresponding author.
